# General practitioners’ reasoning on risk screening and primary prevention of stroke – a focus group study

**DOI:** 10.1186/s12875-018-0883-6

**Published:** 2018-12-04

**Authors:** Ann-Helen Patomella, Gustav Mickols, Eric Asaba, Gunnar Nilsson, Cecilia Fridén, Anders Kottorp, Bo Christer Bertilson, Kerstin Tham

**Affiliations:** 1 Department of Neurobiology, Care Sciences and Society, Karolinska Institutet, Alfred Nobels Alle 23, 141 83 Stockholm, Sweden; 2 Malmo Hogskola, Malmo, Sweden

**Keywords:** Stroke, Primary prevention, Primary health care, Risk management, Qualitative research

## Abstract

**Background:**

By screening and modifying risk factors, stroke incidence can be reduced. Clinical guidelines states that primary prevention of stroke is a responsibility and task of primary health care, but research shows that this not always the case. The aim of the study was to explore and describe what characterizes GPs’ reasoning around risk screening and primary prevention among persons at risk for stroke in primary health care.

**Methods:**

A qualitative design based in a grounded theory approach was chosen in order to investigate this unexplored research area. Data collection was done using focus group interviews and data was analysed using a constant comparative method. Twenty-two GPs were interviewed in four focus groups.

**Results:**

Findings showed that GPs perceived difficulties in prioritizing patients with an unhealthy lifestyle and described a lack of systematicity in their procedures, which complicated their clinical decisions concerning patients with stroke risk factors. The results showed a lack of systematic risk screening methods. Time constraints and the reimbursement system were described as hindering the preventive work.

**Conclusion:**

There is a need for a more proactive, transparent and systematic approach in the distribution of GPs’ time and reimbursement of prevention in primary health care. The findings suggest, by developing new methods and approaches such as digital clinical decision-making tools and by implementing inter-professional team-work, the quality of the primary prevention of stroke could be improved.

**Electronic supplementary material:**

The online version of this article (10.1186/s12875-018-0883-6) contains supplementary material, which is available to authorized users.

## Background

One of the most efficient ways of reducing the incidence of stroke is to reduce the modifiable risk factors for stroke, accounting for up to 90% of the attributable risk [1, 2]. Swedish and European guidelines suggest increased actions to detect and prevent cardiovascular and stroke risk factors [3, 4]. Interventions facilitating lifestyle change in combination with pharmacological treatment are considered most effective in reducing the risk of stroke [1, 5–7]. Major modifiable risk factors for stroke are: smoking, hypertension, diabetes, excessive alcohol consumption, cardiac disease (including atrial fibrillation) and lack of physical exercise, psychosocial factors (stress, depression), elevated waist-to-hip ratio, poor diet and dyslipidemia. Prevention of stroke is most efficiently facilitated as early as possibly, preferably in primary health care (PHC); thus, methods for discovering risk factors are needed in order to initiate early prevention.

The Swedish guidelines for disease prevention propose lifestyle counselling and medical treatment of stroke risk factors [3]. A study in Sweden of first stroke patients [8] showed that most general practitioners (GPs) had intervened on known stroke risk factors before the actual stroke. However, in 75% of the patients with hypertension, actions were taken, but only in 54% where smoking was a risk factor. In Great Britain, only 64% of patients with a calculable risk of CVD or undergoing preventive treatment for CVD were receiving pharmacological treatment according to the guidelines [9]. A study among GPs and cardiologists in Europe [10] revealed that 62% of physicians used subjective risk assessment instead of the proposed guidelines.

There is inadequacy and uncertainty in how physicians intervene in the case of patients at risk of a secondary stroke [11, 12]. We found only one study that focused on the primary prevention and modifiable risk factors of stroke, from Indonesia, a setting without established stroke prevention guidelines [13]. The study suggested that physicians do not consistently screen for stroke risk factors in healthy patients, but rather that patients’ appearance, type of complaint or other concurrent risk factors may prompt a screening. To improve the detection and primary prevention of stroke we need to better understand the clinical reasoning among GPs concerning their detection and treatment of risk factors.

The aim of the study was to explore and describe what characterizes GPs’ reasoning on risk screening and primary prevention among persons at risk for stroke in PHC.

## Methods

### Study design and participants

In-depth focus group interviews were conducted with 22 GPs in PHC. The focus group approach was chosen so as to encourage reflections and discussions to obtain as wide a range of opinions as possible [14], and at the same time to keep the number of sessions to a reasonable level. PHC settings were selected with purposeful sampling in order to obtain diversity and a rich spectrum of information and data [15], representing different socio-economic populations in the Stockholm area. Interviews were scheduled as lunchtime meetings (announced three weeks ahead and open to those interested) in the facilities of the respective health care centre. Written informed consent was received from the participants before the interviews. Ethical approval was given by the regional ethical review board in Stockholm, reference number 2015/834–31/2.

### Data collection

A semi-structured interview guide (Additional file 1) was developed and utilized during focus group interviews. The focus of the interviews was to investigate what characterized the GPs clinical screening and reasoning in relation to prevention of stroke. Questions were modified slightly between focus group sessions in order to explore and build on experiences with the aim of gathering data with rich descriptions of relevance within local contexts of PHC [16, 17]. Examples of questions are: “How do you define a patient at risk for stroke?” and “What preventive intervention do you offer for patients at risk for stroke?”. The first two focus groups were conducted at an urban PHC centre, the third and the fourth at two different suburban PHC centres. Interviews were conducted in Swedish, recorded with digital voice recorders and transcribed verbatim. The length of the sessions varied between 35 and 45 min, and they were moderated by two experienced research team members.

### Analysis

A constant-comparative approach drawing on grounded-theory techniques was utilized [18, 19]. The analytical steps can be characterized as: coding and comparison of incidents, integration of categories and their properties, delimiting of theory, and lastly construction of theory [18, 19]. Firstly, transcripts were read in order to grasp the material [17]. The next step was initial coding, involving re-reading transcripts and going through the data line-by-line [19]. Data was continually coded and compared and memos were written. All codes and memos were grouped into larger clusters and subsequently compared, forming categories. These categories were discussed in the research group, so as to increase the trustworthiness of the results [20]. A theoretical draft was written, to include all different aspects of GPs’ reasoning in relation to the aims of the study, and this resulted in three major categories.

## Results

### Participants

The interviews in the focus groups were carried out between November 2015 and March 2016. The participant’s positions and grade of specialty varied from assistant GP to specialist and operations manager (See Table 1).

**Table 1:** Participant characteristics. Basic characteristics of participating GPs

Characteristics	
Sex: male/female (*n*, %)	11 (50.0%) / 11 (50.0%)
Health centre location: urban/suburban (*n*, %)	10 (45.5%) / 12 (54.5%)
Native language: Swedish/other (*n*, %)	15 (68.2%) / 7 (31.8%)
Median age, range	42, 29–67
Mean years since medical degree, range	16.5, 1–37

### Findings

From the analysis of the focus groups, three categories emerged characterizing the GPs reasoning on risk screening and primary prevention of patients at risk for stroke.

The first category **Situations facilitating or hindering risk detection***,* describes factors and situations that made the GPs screen, and situations when risk screening was not carried out. The second category **A joint understanding of risk and need for change**, describes how the GPs treat and handle patients at risk for stroke. Most risk factors for stroke also are generalizable for CVD and in the interviews; the GPs many times relate their answers to the general risk and prevention of CVD. In the third category, a **Lack of systematicity in clinical routines** summarize how systematicity was or was not present in the screening and prevention of stroke, and illustrate improvements that the GPs wished for. This category was incorporating the first two categories.

#### Situations facilitating and hindering risk detection

GPs reasoning on risk screening was related to the unique situation that emerged in the meeting with the patient, and related to the opportunities available within the clinic. Whether screening was performed on a patient or not came down ultimately to a number of aspects and circumstances that would cause the GP to carry out screening of risk for stroke or part of a screening, for example checking blood pressure. Other circumstances created a situation that that caused the GP to miss an opportunity to screen, even though they suspected risk for stroke.

GPs described several scenarios when stroke risk screening was carried out, for example when a disease was a known risk factor for stroke:

*“And if it’s someone we already know, are in, are having problems that can increase the risk for stroke, we do it [screen], several times per year hopefully, at least twice if it, if you continue at the same rate you still do a risk screening every time they come in.”*

Another scenario described was when suspected risk was based on the patient’s appearance:

*“You might also get a suspicion when you see the patient in the room and you see like maybe, well how it actually feels in the room when you see the patient’s posture and also a bit of their personality as such”.*

The participants described that the patient’s complaint and certain symptoms directed the GP’s thoughts toward increased risk; a complaint of a headache was exemplified as making the GP suspect hypertension. They also described that sometimes an abnormal value appeared by chance (“en passant”) during an examination and the GP wanted to continue investigating:

*“It might be that they have an appointment for something completely different and then we find high blood pressure or that you find atrial fibrillation or [several persons agree] or just “en passant” like that”.*

The GPs described that if there was opportunity and time, they acted on a plausible risk factor, but if time was constrained, they only had the possibility of focussing on the original complaint that the patient had sought care for:

*“If we have the possibility and think that there might be something, we try to get a blood pressure reading or that you listen a little extra sort of… but, sometimes it’s hard because it might be that the appointment is for something else, and you don’t really feel that you have the time”.*

There were, however, GPs that described strategies for coping with the lack of time. If they suspected a risk, but did not have the time to screen on that occasion, they booked in the patient for a new appointment anyway:

*“If you suspect something, you’ll want to book that patient for an [appointment], establish contact if you haven’t seen that patient before, I think [several person agree]”.*

The GPs explained that they had to prioritize their care to the patients due to a lack of resources such as time and how the compensation system was constructed, and thus patients with a high medical risk were prioritized:

*“We work quite a lot with ill patients, and we are quite good at treating them, but we haven’t had as much resources for working with healthy people, those that are in the grey area for prevention, and that has probably been partly because we have had to prioritize differently, and that’s because, nurses have not had so much time [several persons agree] to do such work, but we realize that there is potential; we realize that we could work differently in the future”.*

#### Getting a joint understanding of risk and the need for change

This category presents the GPs’ reasoning on how to prevent stroke using information and understanding as keys to engage the patients in their stroke prevention. The category also includes aspects described as hindering the patients’ participation in their own treatment. Commitment to change was described as a long-term process and that they as GPs could not dictate the patient’s choices:

*“The GP is not a policeman, should not say, “take that,” or “don’t take that,” but we have this knowledge we have to share with them about how it is, and then one has to make one’s own decision”*.

GPs described that, when a patient’s risk for CVD had been confirmed, the GP informed the patient about these risk factors and the importance of treating and improving these factors:

*“Concerning treatment for blood pressure that one doesn’t actually experience as necessary [as a patient], then it is about informing them about the risks of not treating high blood pressure, for example, I always bring up why you treat it; it’s not the blood pressure in itself, it’s the risk, cardiovascular events, so I quite often mention that one wants to reduce the risk for stroke, primary preventive in that case”.*

Some GPs used decision-support and risk assessment tools for CVD as an aid in informing patients of their risk and what they may gain from reducing this risk:

*“Tell your patient like this, potentially if you stop smoking and we manage to get your cholesterol to this level and your blood pressure to the lower interval, what will your risk be, then he calculates a much lower risk and then, see, you had a 25% risk and now you only have a 9% risk, and that is much more effective in convincing them if they see this in a [calculation], because some people want to see in order to understand*”.

In contrast, calculations were also used to inform low-risk patients who were concerned for stroke of their low risk through showing them their risk calculation, in order to calm down the patient and inform them why they did not need treatment.

Sharing a common meeting ground with the patient was described as an important way of arriving at a joint understanding of the risk and need for change:

*“It’s in consultation with the patient [several persons agree]; it’s really individually where the patient is at the moment, what to focus your efforts on, so it won’t work saying that you should always do everything, We’ve tried that before, to put all measures in place, but then, when you don’t have the patient with you on the same page, it doesn’t matter what we say, it’s about seeing, finding the patient”.*

Stroke prevention was described as a long-term and continuous process with the patients and that the patients needed time to commit to reducing their risk factors:

*“It’s like a little information to get them to talk about it; sooner or later a lot of people get very motivated, and like, it can take time, a year later during the annual check-up that they come to”.*

The GPs described that patients at risk for stroke often expressed a lack of motivation/interest in changing their lifestyle, and that they sometimes avoided healthcare. There were also factors that made the patient instantly engaged and motivated for treatment, for example if a patient had suffered a previous stroke or coronary infarction.

The GPs described that bringing up the topic of having a lifestyle that is unhealthy with patients at risk was sensitive, and they feared that the conversation would shame the patient into making them avoid seeking medical advice in the future:

*“There are a lot of people who think we will criticize them as well” the doctor criticizes me” like, because I smoke, it’s stigmatized [several people agrees], then you don’t want to bring that up or talk about it because you will get accused. You feel lousy”.*

Patients may accept and expect their blood pressure to be taken and blood samples, but to talk about alcohol consumption when patients seek help about some other complaint was described as feeling unnatural and charged with shame.

#### Lack of systematicity in clinical routines

The GPs described that the health care system and how it is currently organized effects how they can work with patients at risk for stroke. A lack of systematicity was found in the descriptions of everyday work and how screening and prevention of stroke was conducted. They described a reimbursement system that favours follow-up of chronic diseases over risk screening of unhealthy lifestyle factors. The GPs wished for a more systematic approach to screening and the prevention of stroke by visualizing risks and the possibility of offering intervention to patients at risk for stroke.

The descriptions from the interviews showed an uncertainty about the definition of a risk patient. This complicated the ability to be systematic:

*"It depends on what we call risk, I think; for me this is an enormous group including everything from asking someone to cut their smoking in half or alcohol consumption to reducing blood pressure to better levels…if someone who has a risk in a year, it’s very few, but if it’s a risk in 10 years it’s very many, so it’s, I think it’s a really difficult question”.*

Many of the patients were described as having multiple risk factors, thus making it hard for the GPs to obtain an overview and prioritizing one risk factor:

*“It can be their weight and it can be their blood sugar and it’s the blood pressure and what vascular risk they have,;, it’s not like, one thing, and what should you focus on”*.

Screening was conducted systematically for certain risk patients, this being due to the benefits in the reimbursement system. As an example, risk factors for diabetes patients had to be documented, otherwise the PHC would lose reimbursement. The GPs strived to work systematically by using risk-stratification tools for CVD in individual patients. Although these calculations were not used systematically for all patients, the GPs were aware of the benefits. As an example, CHA2DS2-VASc (a calculator for ischemic stroke risk in patients with atrial fibrillation) was used for assessing risk in patients with atrial fibrillation; for patients only with lifestyle factors, risk calculations were seldom used. Although the present study aimed at stroke risk screening and prevention, the GPs showed that risk screening was part of a larger disease risk stratification and disease prevention, with the exception for the treatment of atrial fibrillation. The distinction between CVD and stroke risk screening and prevention was not be clear in the findings.

The GPs described their work with new and younger healthy patients differently; some GPs usually did a standard battery of tests on new patients seeking care for the first time, including a test for blood pressure, ECG and life-style factors, independent of their level of suspicion (of risk), and in contrast other GPs did not offer this battery. The GPs described that there was an increasing group of younger patients with a fear of stroke that demanded a screening irrespective of whether the GP suspected a risk or not. These young patients were described as navigating the health system in a more effectively and claiming their rights better than elderly patients:

*”…We almost do, in this area and this young group, we almost do too much testing and like, sampling; it’s very rare that there are any abnormal tests when we do them, and it’s like mostly a normal blood pressure, and so on, but sometimes, if they want to, then…”*.

The interviews revealed that the possibility of using referrals to other health care professionals did exist, but with large variations in why referrals were made or not. Some GPs described that they worked close to referral instances and follow up the patient’s results, like “a spider in a web”:

*“If the patient smokes, we send them to a smoking cessation nurse; if the patient has diabetes, we start with everything and then the diabetes nurse takes over; if the patient is overweight, we write a referral for “FaR”[prescribed physical activity]… …and dietician-referrals, so we sit in the centre you could say; we have a lot of resources around us, so that is the way it works, then they come for an appointment and we look at the results”*.

The GPs expressed a need for referrals offering lifestyle interventions.

The GPs themselves requested a more systematic way of working with risk patients, using the computer as a tool:

*”… you could automatically extract data from lab results so cholesterol, data about smoking, when you put it somewhere and then you can combine it and automatically get, for example, a cardiovascular risk assessment for everyone, and then it would maybe be easier for every GP to see at once, now, but even if the patient has an appointment for something else maybe, then you could see still, okay, I see here you have a really high [risk] and you could initiate a possible [treatment]”*.

The GPs perceived a need for information material that the patients at risk could take home. As it is now, the information was described as scattered:

*”…. there actually is a risk that it actually becomes a jungle and you don’t know”*.

GPs wanted to work team-based with risk patients, tailoring care to the needs of each patient:

“*In a perfect world, I would like to have a physiotherapist connected to our office who only had like, body-awareness and like “soft values”, that would be fantastic [-Training customized for] – a body not a diagnosis”*.

## Discussion

Our findings are consistent with an Indonesian study [13]. The Indonesian physicians reported that they did not routinely screen healthy patients, but that patients’ appearance, type of complaint and if they had other concurrent risk factors could prompt a screening, similar to the scenarios found in our study. In both studies, the patients were described as being reluctant to change their habit. Time constrain pressed the GPs in their decisions in our Swedish study and in the Indonesian study [13].

Our study showed that patients with chronic conditions were prioritized over those with only lifestyle risk factors, and this could be explained by the reimbursement system that favours these interventions. A recent study [21] showed that the Swedish PHC choice reform has shaped the provision of health care in PHCs, resulting in a disadvantage for preventive intervention and patients with complex needs, for which there is neither time nor reimbursement. Time constraint was the major factor hindering the preventive work in the present study. The situation at hand many times lead to the fact that the GP did not act on stroke risk factors. The situations where steered by how the PHCs were organised.

Findings showed a lack of systematicity which is in accordance with previous findings that stroke risk patients are not systematically cared for in PHC [8, 9] nor follow-up of stroke [11, 12]. A Swedish study [8] and an UK study [9] showed that many times measures were not taken for treating and reducing known lifestyle risk factors. This may be a result of limited resources for prioritizing lifestyle related risk factors in PHCs, as found in the results of our study. Reducing unhealthy lifestyle factors is an effective way of reducing stroke risk, as shown in several studies [5, 6]; therefore, an intervention aiming at early detection of patients at risk, bringing up the topic of a need for lifestyle change and facilitating lifestyle improvements could be valuable in reducing stroke incidence in the population.

Our study showed that tools used for risk stratification were not used systematically, although these are suggested by European and Swedish guidelines for disease prevention [3, 4]. The GPs suggested an implementation of a digital tool in order to allow a systematic use of risk stratification and treatment options. A digital approach increases adherence to guidelines and the number of patients receiving treatment for risk factors [22–24]. The GPs wanted to be able to visually show the risk and share information with the patient, and this could also improve the difficulties in gaining a common understanding of the risk and the need for change. The American Heart Association/American College of Cardiology has recently developed a digital cardiovascular risk profile that is relevant for both heart disease and stroke [25], however lifestyle related risk factors stroke such as low physical activity and unhealthy diet are not included nor addressed. The Stroke risk scorecard [26] includes lifestyle related risk factors, but the evidence-base is not established yet.

GPs in our focus groups requested a team-based approach to managing patients at risk, and the results showed a fragmented care of patients. Inter-professional team work in PHC may reduce GPs’ time consumption and lack of resources and, in effect, take better care of patients with lifestyle risk factors [27, 28]. A new professional team member, the prevention practitioner, has been introduced to handle the screening and prevention of chronic diseases with efficient results and with the potential to fill a gap in primary health care [27, 28].

### Strengths and limitations

Sampling was carried out in order to get a wider range of perceptions and opinions on the subject, and thus PHCs from different areas in Stockholm were selected. Not all GPs at the centres participated, meaning that other opinions on the subject could have been missed and may potentially compromise the generalisability of the results. The results are given in a Swedish health care context [21] and generalisability to other health care systems might not be possible. The large variation in age range, years since completion of their degree and the equal sex distribution of participants still demonstrate that sampling was comprehensive. Rigorous standards for conducting qualitative studies [20] were used, and authenticity was provided in the quotations from the participants. One limitation related to the use of a grounded theory approach is that we did not apply theoretical sampling techniques and saturation of the data.

### Implication for research and practice

The results showed lack of compliance to clinical guidelines for stroke prevention in the clinical work at the PHCs. The study findings suggests that the clinical situations arising in the meeting between the patient and the GPs where influenced by the organisational structure and reimbursement system at the PHCs and in this study, more specifically the Swedish context. There is a need for more transparency of the responsibility of the prevention task and a modified reimbursement system that favour the prevention of people at risk so that the GPs could prioritize this group of patients and systematically work preventive in PHC. The results highlight the need for new approaches to risk detection, and interventions using new methods and approaches (digital support systems and inter-professional team work).

## Conclusions

The results describe how the GPs reasoned on risk screening and were characterised, by situations that hindered or facilitated the detection of risk factors for stroke. The opportunistic process of risk detection and preventive interventions often seemed to be hindered by time constraints. They describe their communication with the patients as challenging as regards not being able to have a joint understanding with the patients on the risks for stroke and need for change. Lack of systematicity was characterizing GPs screening and primary prevention of stroke.

## Additional file

Additional file 1: Interview guide: Interview guide for focus group interviews. (DOCX 19 kb)

## Abbreviations

CVD: Cerebrovascular Disease
GP: General practitioner
PHC: Primary Health Care
UK: United Kingdom
