# Supplementary material for: General practitioners’ reasoning on risk screening and primary prevention of stroke – a focus group study
Source: BMC Fam Pract. 2018 Dec 4;19:190. doi: 10.1186/s12875-018-0883-6 (PMC6278124; doi:10.1186/s12875-018-0883-6)
Supplement: Supplementary file 1 — Interview guide: Interview guide for focus group interviews. (DOCX 19 kb) [file 12875_2018_883_MOESM1_ESM.docx]

**Interview Guide**

| Section | Types of questions/prompts |
| --- | --- |
| Background | Please, tell us about your current encounters with patients at risk for stroke.  How would you describe a patient at risk for stroke?  What is his/her earlier encounters with health care? |
| Screening | Do you conduct risk screening? How? When?  How do you define a risk for stroke?  What screening methods or tools, if any, do you use? Why? Why not? |
| Clinical guidelines | Do you use any clinical guidelines of decision support? |
| Intervention | What preventive interventions do you offer and why? |
| Communication with patients | Do you inform patients of their risk for stroke? If so, how? Can you give examples of reactions?  Do you inform patients about and discuss preventive interventions? How? |
| Communication with other health professionals | How do you follow-up patients at risk for stroke?  Do you collaborate with other health care professionals? Why? Why not? |
| Use of digital support and needs | Do you use any digital support system in your work with these patients? What are your experiences and needs? |
